# Inward Rectifier Potassium Channels: Membrane Lipid-Dependent Mechanosensitive Gates in Brain Vascular Cells

**DOI:** 10.3389/fcvm.2022.869481

**Published:** 2022-03-28

**Authors:** Maria Sancho, Jacob Fletcher, Donald G. Welsh

**Affiliations:** ^1^Department of Pharmacology, University of Vermont, Burlington, VT, United States; ^2^Department of Physiology, Faculty of Medicine, Universidad Complutense de Madrid, Madrid, Spain; ^3^Department of Physiology and Pharmacology, Robarts Research Institute, University of Western Ontario, London, ON, Canada

**Keywords:** endothelium, smooth muscle, PIP_2_, cholesterol, Kir2 channels, cerebral blood flow, hemodynamic forces

## Abstract

Cerebral arteries contain two primary and interacting cell types, smooth muscle (SMCs) and endothelial cells (ECs), which are each capable of sensing particular hemodynamic forces to set basal tone and brain perfusion. These biomechanical stimuli help confer tone within arterial networks upon which local neurovascular stimuli function. Tone development is intimately tied to arterial membrane potential (V_*M*_) and changes in intracellular [Ca^2+^] driven by voltage-gated Ca^2+^ channels (VGCCs). Arterial V_*M*_ is in turn set by the dynamic interplay among ion channel species, the strongly inward rectifying K^+^ (Kir) channel being of special interest. Kir2 channels possess a unique biophysical signature in that they strongly rectify, display negative slope conductance, respond to elevated extracellular K^+^ and are blocked by micromolar Ba^2+^. While functional Kir2 channels are expressed in both smooth muscle and endothelium, they lack classic regulatory control, thus are often viewed as a simple background conductance. Recent literature has provided new insight, with two membrane lipids, phosphatidylinositol 4,5-bisphosphate (PIP_2_) and cholesterol, noted to (1) stabilize Kir2 channels in a preferred open or closed state, respectively, and (2) confer, in association with the cytoskeleton, caveolin-1 (Cav1) and syntrophin, hemodynamic sensitivity. It is these aspects of vascular Kir2 channels that will be the primary focus of this review.

## Introduction

Cerebral blood flow (CBF) is exquisitely controlled by a complex network of surface/penetrating resistance arteries and a dense capillary network that match local blood cell delivery with tissue metabolic demands (1). This vascular network is primarily composed of smooth muscle (SMC) and endothelial (EC) cells; however, a variety of other cell types including pacemakers, fibroblasts, and pericytes are also known to be present (2). The plasma membrane of vascular smooth muscle (SMC) and endothelial (EC) cells is capable of sensing electrical, neuronal, and/or physical stimuli (i.e., transmural flow and pressure) and transducing them into arterial diameter changes, a fundamental phenomenon for the precise blood flow delivery needed for optimal brain function (3–5). Smooth muscle cell contractility is tightly coupled to intracellular [Ca^2+^], a fundamental second messenger driving myosin light chain phosphorylation and the subsequent extent of cross-bridge cycling (6). The electrical driving force for extracellular Ca^2+^ entry is set by voltage-gated Ca^2+^ channels (VGCCs) which in turn, are critically regulated by the resting membrane potential (V_*M*_) (7). This absolute parameter is defined by the balance of inward depolarizing and outward hyperpolarizing currents in vascular cells. The latter is largely delivered via membrane-embedded K^+^ channels, including voltage-gated (K_*v*_), ATP-sensitive (K_*ATP*_), Ca^2+^-activated (BK_*Ca*_, IK_*Ca*_, SK_*Ca*_), and inwardly rectifying (Kir) channels (8).

In the brain vasculature, Kir2 channels are prominently expressed in SMCs and ECs and exhibit key properties and functions. While Kir2 channels are often viewed as a resting conductance, their unique biophysical signature—inward rectification at V_*M*_ values negative to E_*K*_, activation by extracellular K^+^, and rapid blockade by micromolar Ba^2+^ (9)—has posed them as significant contributors of arterial myogenic tone development and vascular pathology (10–16). Interestingly, numerous studies have reported direct interactions between Kir channels and their surrounding membrane lipid environment which places the channel in an active or silent state (17–23). At a functional level, their distinct properties enable Kir2 channels to contribute to K^+^-induced hyperpolarization, vessel dilation, and force-sensing (4, 21, 22). Particularly, Kir2 channels are noted to respond to specific hemodynamic stimuli, such as pressure and flow, thus contributing to basal tone and CBF control (22, 24).

In this review, we will summarize the major lipid constituents of the cellular plasma membrane, the structural and biophysical features of Kir2 channels, and the link between membrane lipid content and the activity of such ion channels. Thereafter, we will focus on the distinct molecular and mechanical attributes of the vascular plasma membrane, specifically within smooth muscle and the endothelium. This section will include a comprehensive description of mechanosensitive ion channels, cellular routes of force transmission and signaling membrane microdomains. Finally, we will provide the audience with a detailed picture of the unique lipid-protein interactions that confer vascular Kir2 channels the ability to sense mechanical forces (stretch and/or laminar flow), with a primary focus on cerebral vascular tone and perfusion control.

## The (Vascular) Plasma Membrane: A Lipid Bilayer Containing Key Proteins

Every cell in a living organism contains a thick ∼4–10 nm hydrophobic layer which serves as a physical and selectively permeable barrier between two aqueous and heterogeneous environments: the cytoplasm and the outside of the cell. The plasma membrane represents a mosaic of multiple components—including lipids, embedded proteins, and carbohydrates—that are constantly subjected to spontaneous reorganization by rotational or lateral motion (25). Interestingly, the fluidity of the plasma membrane is determined by the ratio of rigid *vs*. fluid lipid components, which in turn depends primarily on the temperature, and the length/degree of unsaturation of phospholipid fatty acids.

### Major Lipid Constituents of Vascular Plasma Membranes

More than 1,000 different species of lipids coexist within the plasma membrane, heterogeneously separated between the two leaflets and giving rise to complex lipid environments at the cell surface (26). These lipids are amphipathic or dual-loving molecules, containing a hydrophilic (“water loving”) and a hydrophobic (“water-fearing”) constituent. The lipid integral components—including phospholipids, cholesterol, and sphingolipids—in addition to being key structural components of the cell membrane, also interact with other non-lipid membrane constituents such as proteins. Specifically, a wide variety of ion channels, including the strongly inward rectifier potassium (Kir2) channel, require a specific local lipid microenvironment to properly function (22).

Phospholipids—built on a glycerol backbone, two fatty acyl chains and a polar head group—account for the most part of the membrane, accordingly termed phospholipid bilayer. Five types of phospholipids are found asymmetrically distributed across the membrane, including phosphatidylcholine, phosphatidylethanolamine, phosphatidylserine, sphingomyelin, and phosphatidylinositol. The latter group, while making up a minor fraction of cell phospholipids in comparison to the other families, represent key molecules involved in crucial cellular processes due to their ability to interact with a variety of integral proteins in the plasma membrane (27). Phosphatidylinositol and related phosphoinositides (PtdIns) contain negatively charged head groups and are non-uniformly distributed in the inner (cytosolic) leaflet of the membrane. Phosphatidylinositol 4,5-biphosphate (PIP_2_), which has emerged as a highly versatile player in cell signaling, constitutes only ∼1–3% of the phospholipids in eukaryotic cell membranes (28) and represents the most abundant of the doubly phosphorylated PtdIns species (>99%) (29). Measurements of local PIP_2_ concentrations in a cell are still imprecise, however, studies employing fluorescently tagged pleckstrin homology (PH) domains—which bind specifically to membrane PtdIns—have more accurately estimated its effective concentration to range from 2 to 30 μM (30).

Membrane PIP_2_ levels seem to be controlled by a concomitant interplay between breakdown and synthesis. PIP_2_ is synthetized by its precursor phosphatidylinositol-4-phosphate (PI4P) in two successive phosphorylation steps by the enzymes PI4 and PI5 kinases (PIK4 and PIK5). Moreover, the efficient hydrolysis of PIP_2_ provides the source of two important secondary messengers in the cell, inositol trisphosphate (IP3) and diacylglycerol (DAG). Structurally, PIP_2_ contains an inositol head group with an average orientation of 45° with respect to the bilayer surface (31), a phosphoglycerol backbone, and two acyl chains. The net charge of this key signaling phospholipid is believed to be -4, however, it may range from -3 to -5 depending on multiple factors, including the local pH and its interactivity with proteins (32, 33). Despite being a minority phospholipid of the membrane, a large list of ion channels and transporters are reported to require certain levels of PIP_2_ in the membrane to function correctly, thus acting as a local switch with the ability to control regional cellular activity (34–42).

Cholesterol (C_27_H_46_O) is a central lipid in mammalian cells involved in multiple cellular processes at the vascular plasma membrane. It accounts for up to 50% of total membrane lipid molecules, as more than 90% of total cellular cholesterol is confined to the plasma membrane (43). The cholesterol molecule contains four hydrophobic hydrocarbon rings and a weakly hydrophilic hydroxyl (OH) group attached to one end, conferring the molecule an amphipathic character. The OH group facilitates the orientation of the molecule—with the hydrophobic regions pointed toward the hydrophobic core of the membrane—and the interaction with water molecules or adjacent phospholipids, restricting their motion and modifying the intrinsic properties of the lipid bilayer such as fluidity, stiffness and permeability (44–46). Like PIP_2_, cholesterol binds to multiple transmembrane proteins including different types of ion channels (K^+^, Ca^2+^, Na^+^, and Cl^–^ channels), altering their conformational states and regulating their activity (47–51). Cholesterol is additionally involved in determining the physical integrity and functionality of lipid rafts—specific low-fluidity nanoscale domains which function as anchors for residing membrane proteins, representing “signaling hotspots” for key cellular signaling and trafficking events and host-pathogen interactions (52–54).

The cholesterol content in the membrane is defined by the dynamic balance between *de novo* biosynthesis from acetyl coenzyme A in the membrane of the endoplasmic reticulum, receptor-mediated uptake via plasma lipoproteins, transport to the membrane by caveolins, export and storage. These processes are tightly controlled by specific feedback mechanisms to ensure appropriate cholesterol homeostasis and maintain its levels within a narrow range. Therefore, disturbed cholesterol balance may profoundly alter cell/tissue function, lead to cell death, and further contribute to not only severe cardiovascular pathologies—including acute thrombosis and vascular occlusion preceded by hypercholesterolemia and atherosclerosis (55)—but also multiple types of cancer. Interestingly, cholesterol accounts for a large portion of the adult brain (∼35 g), which contains approximately 25% of the total amount of the cholesterol present in the body, making it the most cholesterol-rich organ (56). This fact obviously implies that a dysregulation of brain cholesterol levels or its turnover could contribute to the development or progression of several neurodegenerative disorders (i.e., Alzheimer’s disease or multiple sclerosis) and may represent a target for potential therapeutic avenues.

Despite being less abundant, sphingolipids represent another class of bioactive membrane lipids. The backbone of sphingolipids is ceramide, a molecule composed of a sphingoid head, generally sphingosine—an amino alcohol that contains a long hydrocarbon chain—with a long-chain fatty acid linked via an amine bond. Sphingolipids are a particularly diverse lipid family which regulate structural characteristics of the membrane such as curvature and thickness (54), participate in important intracellular signaling pathways and, mediate dynamic interactions between cells and their surrounding extracellular microenvironment (57, 58). Furthermore, sphingolipids can interact with cholesterol constituting highly dynamic raft-like membrane domains which facilitate the interplay with membrane proteins (54). Particularly, the association of these cholesterol-sphingolipids enriched domains with the protein caveolin constitutes caveola, small (50–100 nm) flask-shaped invaginations of the plasma membrane considered as local hotspots for lipid- and protein-mediated signaling cascades with a key role in mechanosensation in vascular endothelial and smooth muscle cells (59).

### Inward Rectifier K^+^ Channels: Key Membrane Proteins Modulated by Specific Lipid Interactions

**FIGURE 1:**
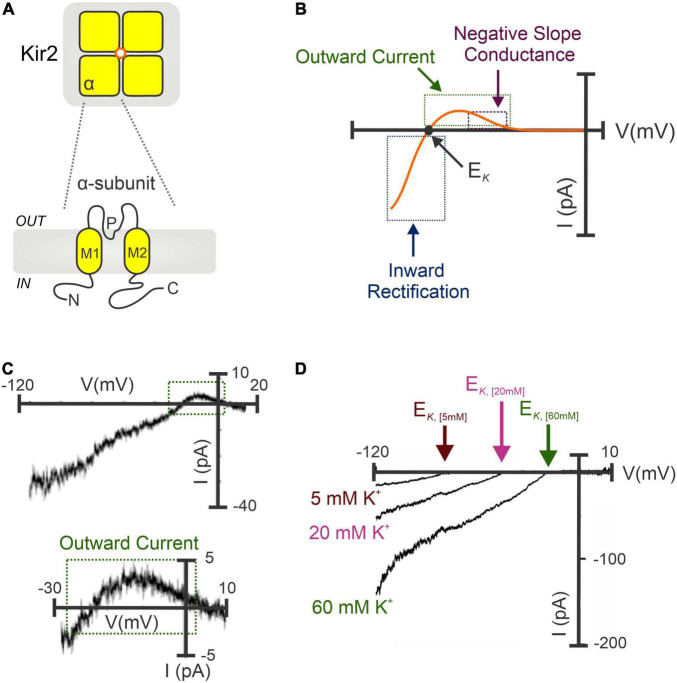
Molecular architecture and biophysical signature of strongly inward rectifying K^+^ (Kir2) channels. **(A)** Kir2 channels constitute tetramers of α-subunits (*top*). Each subunit contains two transmembrane domains (M1 and M2), a K^+^ selectivity pore (P) region, and C- and N-terminal domains (*bottom*). **(B)** Illustrative I-V relation of the Kir2 conductance involving Kir2.1 and/or Kir2.2 subunits. Notably, Kir2 whole-cell currents exhibit the characteristic inward rectification at hyperpolarized voltages (negative to the E*_*K*_*), and a small outward current with a negative slope conductance region at physiological V_*M*_. **(C)** Barium-sensitive Kir2 currents recorded in isolated cerebral SMCs exposed to high extracellular [K^+^] (60 mM) (top). A tiny (<5 pA) outward current with a negative slope conductance was successfully recoded (bottom, magnified from top). **(D)** Potentiation of Kir2 currents when raising the extracellular K^+^ concentrations, which increases Kir2 inward current density and shifts the channel activation curve to more positive V_*M*_, with a subsequent rightward shift in the E*_*K*_*. **(C,D)** Modified from Wu et al. (24) and Smith et al. (61).

Classical strong Kir channels are integral membrane proteins highly expressed in vascular cells from the cerebral (12, 24, 60, 61), coronary (61), mesenteric (62, 63), and renal (64) circulation. Structurally, Kir2 channels constitute tetramers of α-subunits (Kir2.1-2.4) sharing ∼50–70% of amino acid identity (65–67), and each containing two transmembrane domains (M1 and M2), an intracellular N- and C-terminus, and a highly conserved extracellular loop that serves as a K^+^ selectivity filter (Figure 1A), essential for channel regulation and the mechanism of inward rectification (68, 69). This fundamental property (originally defined in skeletal muscle as “anomalous rectification”) arises due to the intrinsic ability of inward rectifiers to conduct large K^+^ currents inward (at V_*M*_ negative to the E_*K*_) while passing small outward currents at more depolarized voltages (24, 61). The inward rectification occurs because of the robust voltage-dependent pore blockade by impermeant intracellular cations mainly polyamines and Mg^2+^ at depolarized membrane voltages, whereas at hyperpolarizing V_*M*_ the blocking cations are absent from the pore, enabling K^+^ to flow (65, 69). Despite being inward rectification the biophysical fingermark of Kir channels, the small (a few picoamperes of amplitude) outward current is largely important for controlling arterial V_*M*_, basal tone and blood flow. This tiny outward current displays a negative slope-conductance region at physiological voltages (66, 70, 71) that increases other outward K^+^ currents to boost the original hyperpolarizing/vasodilatory response (61, 62, 72), playing thus a fundamental role in the regulation of regional tissue blood flow and flow distribution (Figures 1B,C). This unique electrophysiological property is predominant in channels containing Kir2.1 and Kir2.2 subunits—elements robustly expressed in vascular SMCs and ECs (22, 24). Moreover, Kir channel activity relies on extracellular [K^+^] levels. Particularly, increases in [K^+^]_*o*_ leads to an augmentation of Kir inward current and shifts the channel activation curve to more depolarized potentials and, a subsequent rightward shift in the E_*K*_ (24, 73) (Figure 1D). In a physiological scenario, vascular cells display membrane potentials positive to the E_*K*_, this implies that a rightward shift in the channel activation curve may also enhance the amplitude of the outward current. Due to this particularity, Kir channels are poised to contribute to K^+^-evoked functional hyperemia in excitable tissues (i.e., brain or skeletal muscle), a fundamental process that ensures regional increases in blood flow in to maintain tissue function and health (12, 73, 74). Micromolar concentrations of extracellular BaCl_2_—a potent and selective voltage-dependent Kir channel blocker—represent an ideal tool to characterize the molecular basis of Kir2 currents in vascular tissues (4). Barium sensitivity substantially differs depending on the subunits that conform the Kir2 channel. For instance, heteromers of Kir2.1-Kir2.2 subunits (robustly expressed in the vasculature) exhibit IC_50_ values ranging from 0.7 to 5 μM (at -100 to -120 mV), different from that of homomeric Kir2.1 or Kir2.2 channels (IC_50_ = 16 μM and 2.3 μM for Kir2.1 and Kir2.2 at -120 mV, respectively) (21). The selectivity of BaCl_2_ to block Kir channels is well-validated by two important facts: (1) Ba^2+^ exclusively blocks the inward rectification at voltages more hyperpolarized than E_*K*_ and, (2) tissues expressing Kir channels exclusively exhibit [K^+^]-induced vasodilation sensitive to Ba^2+^ (24, 61). Consistent with this, Ba^2+^ superfusion caused a pronounced depolarization and subsequent vasoconstriction of isolated intact arteries, with this effect being greater at low intravascular pressures when resistance vessels exhibit hyperpolarized V_*M*_ values and limited myogenic tone (23, 60). Accordingly, *in vivo* studies in rodents have shown a potent BaCl_2_-induced vasoconstriction of brain surface (14) and cremaster arteries (75).

**FIGURE 2:**
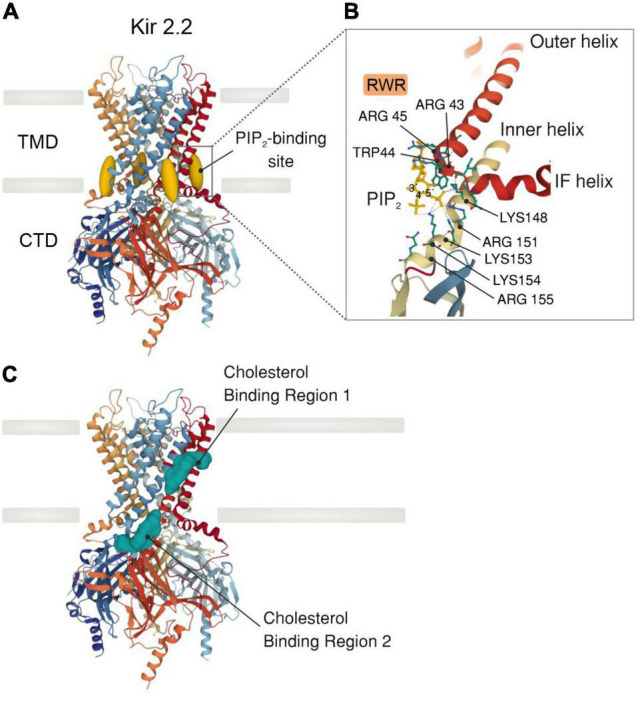
X-ray crystal structure of a Kir2.2 channel with PIP_2_ or cholesterol bound. **(A)** Side view of a model of a Kir2.2 tetramer consisting of a transmembrane domain (TMD), which comprises the potassium-selective pore, and a large cytosolic (CTD) domain (PDB ID: 3SPI). PIP_2_ molecules (dark yellow) bind at the interface between the TMD and the CTD. **(B)** Detailed illustration showing the PIP_2_-binding site in a similar orientation as outlined in A. The primary amino acid residues bound (dashed lines) to PIP_2_ are highlighted. All side chains are shown as solid lines. **(C)** Side view of a Kir2.2 channel model illustrating the two distinct cholesterol binding regions (green): one residing in the center of the TMD and the other at the interface between the TMD and the CTD.

**FIGURE 3:**
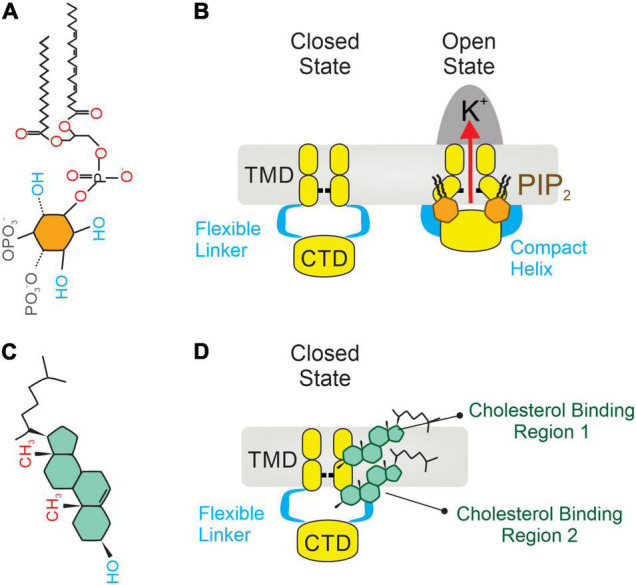
Two integral membrane lipids, PIP_2_ and cholesterol, bind to and regulate the activity of Kir2 channels. **(A)** Molecular architecture of a PIP_2_ molecule, constituted by an inositol polar group, a phosphoglycerol backbone and two acyl chains. **(B)** Mechanism of Kir2 stimulation by membrane PIP_2_ binding, which causes a change in the architecture of the flexible linker into a compact helix. Then the cytoplasmic domain (CTD) translates toward and becomes tethered to the transmembrane domain (TMD), which in turn mechanically opens the channel. **(C)** A cholesterol molecule contains three main parts: (1) tetracyclic carbon ring, (2) a polar 3β-hydroxyl (OH) group, and (3) a short non-polar carbon chain. **(D)** Location of two non-annular hydrophobic cholesterol binding domains in Kir2 channels: one located in the center of the TMD (1), and the second at the interface between the TMD and the CTD (2). Upon direct cholesterol interaction, the hinging motion of the transmembrane pore helix changes, causing channel stabilization in a preferred closed (silent) state.

Inward rectifiers were classically considered poor regulatory targets (62, 76), however, recent collecting evidence has shown that Kir2 channel activity is modulated by the surrounding membrane lipid environment. This includes two signaling lipid regulators with opposing effects, PIP_2_ and cholesterol, which stimulates and suppresses the Kir2 channel activity, respectively. Specifically, PIP_2_ is required at certain levels in the plasma membrane to stabilize Kir2 channels in a preferred open (active) state, and transient depletion of PIP_2_ by PLC activation *in vivo* suppresses these functions (17, 39). In this sense, sophisticated crystallographic and functional analysis have provided a detailed structural depiction of the binding sites by which negatively charged phosphates of PIP_2_ electrostatically interact with positively charged (basic) amino acid residues (arginines, lysines, and possibly histidines) of Kir2 channels to directly modulate their activity. In particular, the majority of these basic amino acid residues are positioned at the interface between the transmembrane and the cytosolic domains of the channel allowing PIP_2_ to interact with both regions (Figure 2A). Specifically, the inositol polar group of the phosphoinositide makes interactions with a specific phosphatidylinositol region in the cytoplasmic domain of the channel whereas a conserved non-specific phospholipid binding site (RWR; containing the amino acid residues ARG43, ARG45, and TRP44) located in the transmembrane domain (N-terminus of the outer helix) provides specificity for the acyl chains and phosphopglycerol backbone of PIP_2_ (39, 77) (Figure 2B). Nonetheless, the only crucial element of PIP_2_ for Kir channel regulation seems to be the number of phosphates in the polar head as other PtdIns species (PtdIns(4)P and/or PtdIns) exhibited diminished activity or failed to stimulate Kir2 channels (39). In this sense, various polyvalent cations including polyamines, trivalent metals, neomycin and polylysine are capable of blocking Kir channel activity by screening the negatively charged polar groups of PIP_2_. This membrane lipid binds to the channel and provokes a conformational change to open the gate, thus resulting in increased channel activity. Particularly, this conformational change includes the contraction of a peptide flexible linker into a compact helical structure which induces a reorientation and rotation of the cytoplasmic region, becoming tethered to the transmembrane domain which in turn, provides a tangential force that automatically opens the gate of the channel and promotes the conductance of K^+^ ions across the pore (77) (Figures 3A,B). A Previously published electrophysiology study employing Kir2.1 (monomeric, dimeric, or tetrameric) constructs expressed in *Xenopus* oocytes showed that only one subunit of the tetrameric arrangement of the Kir channel is sufficient for channel activation to its fully open state. Additionally, further interaction with additional subunits induces multilevel positive cooperativity, increasing channel availability to open (reducing dwell time) and facilitating channel conductance (78). Among the diverse Kir subunits, Kir2.1 channels possess high affinity for PIP_2_, and are not influenced by its depletion. In contrast, other subunits including Kir2.2 and Kir2.3 display relatively low PIP_2_ affinity and therefore are robustly influenced by PIP_2_ breakdown (79). Moreover, the strength of PIP_2_-Kir direct interactions not only enhances Kir channel activity but also controls the sensitivity of the channel to other regulatory factors such as pH, protein kinase C, Mg^2+^ or phosphorylation (79).

In the brain circulation, our recent study (22) revealed that ECs, freshly isolated from third-order middle and posterior cerebral arteries, possess Kir2.1-Kir2.2 heterotetramers which are tightly controlled by membrane PIP_2_ levels. In this context, EC Kir2 conductance was impacted in a timely manner by dialyzing (conventional whole-cell patch-clamp configuration) the cells with diverse pharmacological drugs that modify membrane PIP_2_ concentrations. Consistent with PIP_2_ facilitating Kir channel activity, depletion of this signaling lipid by dropping the intracellular ATP concentration to zero, blocking PIK, or adding neomycin—an aminoglycoside that impairs the electrostatic interaction between PIP_2_ and the Kir channel—to the patch pipette induced an evident steady decrease of the Kir current. This whole-cell Kir current rundown was further prevented by incorporating the water-soluble short-chain analog of PIP_2_ (dioctanoyl-PIP_2_, diC8-PIP_2_) to the intracellular solution, a finding that poises membrane PIP_2_ as an intrinsic regulator of endothelial Kir2 channels by promoting channel stabilization in a preferred open (active) state. Surprisingly, Kir2 channels residing in SMCs were insensitive to these pharmacological manipulations over time, suggesting that membrane PIP_2_ is a minor modulator of this channel pool (22). These findings are consistent with the coexistence of two cellular populations of Kir2 channels (ECs *vs.* SMCs) in cerebral resistance arteries which are distinctly regulated by their neighboring lipid microenvironment.

As the brain vascular tree ramifies, pial (surface) arteries give rise to penetrating and parenchymal arterioles, and eventually to a capillary network containing hundreds of capillaries which vastly extend the arteriolar territory of perfusion. Capillaries, the narrowest blood vessels in the body, are tubes made up of a single layer of ECs which act as a neural activity-sensing network being thus much more than simple conduits for blood (12). At the capillary level, and consistent with our work, PIP_2_ has been reported as a requisite molecule to maintain EC Kir2.1 channels in a preferred open state. This property makes it essential for the generation of retrograde electrical signals from capillaries which dilate upstream arterioles and contribute to the fundamental process by which CBF increases to satisfy the metabolic demands of active neurons (functional hyperemia) (23). Since PIP_2_ is also known to exert opposite effects on capillary EC TRPV4 channel activity, this minor lipid has been suggested to serve as a regulatory switch that actively governs the balance between electrical (Kir2.1) and Ca^2+^ signaling (TRPV4) (80). In this context, Dabertrand and colleagues have recently revealed that exogenous application of PIP_2_ corrected CBF deficits by recovering capillary EC Kir2.1 activity in a monogenic model of Small Vessel Disease (SVD)—the major cause of stroke and dementia (15).

Contrarily to PIP_2_, membrane cholesterol strongly suppresses the activity of Kir channels by placing the channel in a preferred closed (silent or inactivated) state (81). This notion originated from electrophysiology studies that described a reduction in whole-cell Kir2 currents—being that the channel open probability, unitary conductance and channel protein expression were slightly impacted—when membrane cholesterol levels were increased (46). The molecular foundation of cholesterol regulation of Kir channels was effectively elucidated by the Levitan group which identified several cytosolic residues in the G-loop, the N-terminus and the peptide linker between the C-terminus and the inner transmembrane helix of the Kir2.1 channel. These residues form a belt that embraces the cytosolic pore and controls the cholesterol sensitivity of the channel. The cytoplasmic belt is different from the cholesterol binding site, but it allows cholesterol binding to a different part of the channel which is critical for the functionality of the gating system (82). Paradoxically, this cytosolic arrangement also contains some essential residues for PIP_2_ sensitivity, a fact consistent with both membrane lipids (PIP_2_ and cholesterol) sharing a common regulatory hub that controls the Kir2.1 channel state (open *vs.* silent). Interestingly, this attribute is absent in Kir2.2 channels as they lack the aforementioned overlap in PIP2- and cholesterol-binding residues (164). While cholesterol mediates Kir2 activity by binding to these specific residues, it has been also proposed to indirectly impact ion channel function during changes in the physical properties of the membrane (i.e., membrane stiffness). In this sense, the substitution of native cholesterol with its optical isomer (epicholesterol) not only abrogated the inhibitory effect, but also exerted opposing effects on endothelial Kir2 channel activity, revealing that the sensitivity of Kir2 to cholesterol is stereo-selective to cholesterol optical analogs (83). These findings effectively support the hypothesis that cholesterol suppresses Kir channel activity directly rather than by altering plasma membrane structure (84). In this context, Kir2 channels contain two non-annular cholesterol-binding motifs, one of which resides in the center of the transmembrane region and the second at the interface between transmembrane and cytoplasmic domains of the channel (Figure 2C). Upon favorable cholesterol-channel binding, the hinging motion of the transmembrane pore helix is altered, promoting thus channel stabilization in a preferred closed state (20) (Figures 3C,D).

Further studies using endothelial culture models revealed that Kir2.1 and Kir2.2, the major structural components of the endothelial Kir channel, are suppressed or activated following enriching or depleting membrane cholesterol, respectively, whereas Kir2.3 and Kir2.4 were less sensitive to cholesterol content manipulations (51). Moreover, the degree of Kir2 suppression matches with the amount of membrane cholesterol loading, being completely reversible following subtraction of cholesterol excess. The negative effect of cholesterol on EC Kir activity was also demonstrated *in vivo*, where Kir2 currents recorded in ECs isolated from the aorta of Yorkshire pigs fed atherogenic high-cholesterol diet were significantly diminished comparing to those recorded from healthy animals (85). In an attempt to translate these key findings into the brain vasculature, investigators have dialyzed native SMCs and ECs with methyl-β-cyclodextrin (MβCD), a compound widely used in cell biology to deplete cells of cholesterol (22). This pharmacological manipulation impacted Kir2 channel activity in a time-dependent manner, an effect that was subsequently prevented when the cells were exposed to cholesterol-saturated MβCD. Intriguingly, these responses were particularly evident in SMC Kir currents comparing to the endothelium, identifying cholesterol as a major modulator of the smooth muscle Kir channel population by promoting its stabilization in a preferred closed state.

## The Plasma Membrane of Vascular Cells: An Efficient Mechanical Sensor

Resistance arteries are small blood vessels (<400 μm in diameter) that contribute to peripheral vascular resistance. The vascular wall of these blood vessels displays structural and intrinsic (local) responses to ever-dynamic forces to eventually regulate vascular tone and match blood flow to tissue metabolic demands. This ability is accomplished by the plasma membranes of two of the principal constituents of the vascular wall—smooth muscle and endothelium—which are constantly exposed to mechanical stimuli, mainly originated by a pulsatile blood flow—stretch (tensile stress) and/or shear stress (86). In this context, specific mechanosensitive molecules residing within the plasma membrane—ion channels, caveolae, and/or surface receptors—can modify their conformational state and electrical/chemical properties in response to mechanical disturbances and transduce them into a physiological response (87, 88).

### Stretch and Myogenic Tone

The vascular smooth muscle layer of a resistance artery (or of downstream penetrating arterioles) possesses an inherent ability (independently of the endothelium and nerves) to contract—and reduce the luminal diameter—in response to an abrupt increase of transmural pressure (i.e., radial stretch) (89). In the brain, this phenomenon known as “myogenic tone” elevates resistance to blood flow (90, 91), and is essential for setting basal vascular tone, maintaining a constant perfusion over a range of intraluminal pressures, and fine-tuning local CBF while protecting downstream capillary networks from damage (6, 92). Interestingly, myogenic tone responsiveness becomes more robust as vessel size decreases (93, 94), and this could be explained by an increase in vessel wall distensibility as the vascular tree ramifies (95). Moreover, the myogenic tone of resistance arteries can be influenced by hemodynamic forces (i.e., flow), metabolic factors and vasoactive mediators released from other cell types, including the endothelium (96–98).

The underlying mechanisms of this phenomenon have been extensively studied over previous decades (90, 99–103). An increase of intraluminal pressure modulates the activity of stretch-sensitive ion channels expressed in SMCs resulting in membrane depolarization and the activation of voltage-gated calcium channels (VGCCs), which in turn elevates the intracellular levels of contractile Ca^2+^ (7, 104). As a result, mechanosensitive ion channels residing in SMCs are often investigated due to their ability to serve as pressure sensors by depolarizing arterial membrane potential (V_*M*_) and initiating the myogenic response (90, 105). In the brain, the myogenic response is imperative for precise vascular SMC function and accordingly, myogenic tone disruption has been associated with many vascular diseases including stroke, hypertension and dementia (106–108).

### Shear Stress-Induced Vasodilation

The endothelial layer that lines the vasculature is experiencing a continuous frictional drag force of the blood flow and by rolling blood cells over its luminal surface. This type of internal stress known as (fluid) shear stress is opposed by tension and deformation in the endothelium and initiates a complex signaling cascade—involving mechanosensory complexes containing multiple surface molecules including ion channels and integrins—that in short timescales causes vessel dilation to preserve the functionality of the vasculature (109). ECs are exposed to diverse shear stress patterns *in vivo* that may vary in magnitude, direction and temporal characteristics in relation to their location within the vascular bed.

Although the exact signaling pathways that facilitate endothelial responses to flow are incompletely determined, mechanosensitive channels have been suggested as major sensors of laminar flow, being able to respond to changes in mechanical loading, integrate the signal and elaborate a response that impacts the ion channel permeability of the plasma membrane. A large number of mechanically activated calcium and potassium channels including P2RX4 (110), transient receptor potential (TRP) vanilloid subtype (TPV4) (111), Piezo 1 (112), Kir2.1 (113), and small-conductance Ca^2+^-activated K^+^ channels (SK) (114) have been suggested to play a role in endothelial responses to blood flow in various vascular beds (109). In the brain, endothelial cells express a complement of K^+^ channels including Kir, K_*ATP*_, intermediate-conductance Ca^2+^-activated K^+^ channels (IK), and SK (11, 21, 61, 108), and other ion channel families such as TPV4 (80, 115) or Piezo1 (116). Notably, the expression of these ion channel species displays some differences depending on the anatomical location of the ECs along the vascular tree. Arterial/arteriolar ECs possess IK and SK channels that play a critical role in transducing rises in intracellular Ca^2+^ levels into hyperpolarizing/dilatory signals, whereas this ion channel family is absent in the capillary endothelium. Notably, ECs do not express voltage-gated channels; however, these cells are electrically coupled to SMCs and thus able to directly govern vascular basal tone (117).

### Mechanosensitive Ion Channels, Cellular Routes for Force Transmission, and Signaling Microdomains

Mechanosensitive ion channels represent a firmly established set of biological molecules which possess an extraordinary capacity to sense and process external mechanical stimuli by initiating an electrical or chemical signal, eventually integrated into a coherent cellular response (118). Among them, several ion channels, including TREK-1 (119) and TRPC1 ion channels (120), are directly activated upon force transmission through the lipid bilayer without the involvement of any additional associated cellular components (i.e., cytoskeleton), a type of gating model typically observed in bacterial ion channels known as the “bilayer mechanism.” In contrast, the vast majority of mechanosensitive ion channels residing in animal cell membranes—including many TRP channels (121–125)—are governed by the “tethered mechanism” by which force is indirectly applied to them via cytoskeletal, extracellular matrix (ECM) molecules (e.g., fibronectin) and, accessory scaffolding components (39, 70). As a wide range of physical signals impact the plasma membrane, they are transferred across the ECM to the cytoskeleton (“dual-tether model”) through modulating spanning integrin receptors at focal adhesion sites (126). Therefore, cytoskeletal actin and additional structural components may represent key routes of cellular force transmission with clear impact on ion channel mechanosensitivity.

Actin is the most abundant cytoskeletal protein in vascular smooth muscle (∼20% of total protein content) with a critical role in the maintenance of cellular integrity (127), whereas contractile actin filaments associate with myosin to form the contractile machinery (128). The architecture of the actin cytoskeleton in SMCs is a dynamic process, switching from polymerized to depolymerized states and contributing to muscle contraction or relaxation, respectively. This process is mainly defined by the filamentous- (F) to globular- (G) actin ratio which under resting conditions—containing a significant pool (∼30%) of G-actin monomers—is approximately 2:1 (129). An external mechanical stimulus (pressure/stretch) provokes the transition of G- into F-actin leading to smooth muscle contraction and an increase in force generation. Moreover, studies using isolated and pressurized cerebral arteries demonstrated the importance of this ever-dynamic process for myogenic tone development as actin cytoskeletal disruption blunted pressure-induced constriction of cerebral arteries (129). In the context of ion channel function, pressurization of cerebral arteries treated with actin polymerization blockers (i.e., cytochalasin B) elevated Ca^2+^ influx and membrane depolarization. However, the precise identification of the ion channel species and scaffolding protein intermediates involved in this process remain undefined (130). In this context, members of the dystrophin-associated protein complex (DAPC) have been studied, as they mainly operate as connectors of the actin cytoskeleton to the membrane and ECM (131).

Dystrophin is a large (427 kDa; 110 nm) sub-membrane cytoskeleton protein that interacts with syntrophin and various protein elements to constitute the DAPC in vascular smooth muscle (131). Loss of function in the *DMD* gene encoding Dystrophin causes Duchenne muscular dystrophy, a rare but distinctive disease characterized by progressive muscle weakness and wasting primarily in males. The severe pathological effects of this rare genetic disease have stimulated a solid focus of research exploring the function of dystrophin and associated proteins (132, 133). Dystrophin spans actin filaments laterally, binding alongside to them through the N-terminal domain and forming a solid physical bridge with the cytoskeleton (134). The C-terminus of the dystrophin molecule enables the assembly of the DAPC by providing multiple binding sites for a group of signaling and channel proteins including syntrophins, sarcoglycans, dystroglycans, and dystrobrevins (131, 135). Collectively, the DAPC anchors the actin cytoskeleton to the ECM to physically protect the sarcolemma from the membrane perturbations developed during muscle contraction (136). Consequently, the lack of dystrophin increases the chance of sarcolemmal rupture following skeletal muscle contraction, a characteristic feature of Duchenne muscular dystrophy (136). In the vasculature, the loss of dystrophin in SMCs leads to decreased vessel contractility and impaired stretch-induced gene transcription, resulting in overall smooth muscle function and mechanosensitivity decline (137).

Syntrophins (from the Greek, syntrophos, thought to mean companion) are 58 kDa membrane-associated adaptor proteins involved in DAPC formation and the recruitment and regulation of surrounding signaling proteins—including ion channels—into a regulatory macromolecular assembly (138). The syntrophins are a multigene family involving five homologous isoforms (α1, β1, β2, γ1, and γ2) that display tissue-specific expression profiles. Among them, α1 is predominantly abundant at the sarcolemma of muscle fibers and directly binds to the C-terminal domain of dystrophin (139–141). Syntrophins lack intrinsic enzyme activity, however, they contain unique domains than enable them to bind other proteins and arrange signaling microdomains at the cell membrane. Structurally, all isoforms possess two pleckstrin-homology (PH) domains, one PDZ domain that facilitates protein-to-protein interactions, and a syntrophin unique (SU) domain located in the C-terminus which directly binds to dystrophin (140). Particularly, the PDZ domain recognizes and attaches to protein targets containing the C-terminal motif Ser/Thr-X-Φ-COOH, where X and Φ represent interchangeable and hydrophobic residues, respectively (142). Among the protein binding partners, syntrophins haven reported to recruit Kir2 channel subunits via their C-terminal PDZ binding motif (Ser-Glu-Ile) (143). This interaction enables syntrophins to connect Kir2 channels directly to the DAPC and form a structural link with the cytoskeleton. It has also been observed that this interaction is more strongly associated with the Kir2.2 subunit, with weaker interactions reported with Kir2.1 (143). While our understanding of the physiological relevance is still in its infancy, proteomic analyses have identified Kir2-synthrophin interactions in heart, skeletal muscle and brain (143, 144).

The clustering of signaling components with their protein targets—which facilitate rapid cellular responses—is mostly achieved by caveolae (or “little caves”) (145). Caveolae are unique cholesterol-enriched, flask-shaped and small (50–100 nm in diameter) membrane invaginations with a major role in many cellular processes including the regulation of cellular signaling cascades and adaptation to the constant tensional changes that cell membranes undergo (146). The curved structure of caveolae is shaped and stabilized by the insertion of caveolin proteins into the membrane. Among them, caveolin-1 (Cav1) and caveolin-2 (Cav2) are widely expressed across multiple tissues and cell types, whereas caveolin-3 (Cav3) is expressed primarily in smooth, skeletal and cardiac muscles (147, 148). Despite the three isoforms that have been identified in vascular smooth muscle, Cav1 is the only component that is required for caveolae formation and its deletion has been shown to weaken contractile responses (147, 149). In fact, the lack of this isoform leads to impaired endothelium-dependent relaxation, contractility and myogenic tone (149). Functionally, Cav1 and caveolae enhance signaling cascades by engaging key molecular components such as ion channels, adaptor proteins, and receptors within microdomains of the membrane (145). Moreover, Cav1 is known to regulate the activity of numerous ion channels and enzymes. As an example, swelling activated Cl^–^ currents are suppressed in Cav1-deficient cell lines but restored by transient expression of Cav1 (150).

Actin filaments are known to associate with and stabilize caveolae, as depolymerization of the cytoskeleton leads to migration of Cav1 throughout the cell (145). This association creates a highly efficient mechanosensitive territory, providing a local area where mechanical stimuli can be focused, sensed, and processed. This mechanism has been proposed for stretch-activated Ca^2+^ channels as they reside in caveolae and their activity is tightly regulated by the actin cytoskeleton (145, 151, 152). Cav1 has been previously reported to directly bind Kir2 channels reducing current density without impairing the single channel properties or membrane protein expression. In this context, Han and colleagues previously demonstrated that Cav1 acts as a negative regulator of Kir channel activity and that Cav1 and cholesterol stabilized the channel in a closed silent state by a shared regulatory mechanism (153) although Cav1 is not required to confer cholesterol sensitivity to the channel. With the aid of crystallography this study effectively identified two putative Cav1 binding domains, the first one at the interface between the outer transmembrane helix and the N-terminus, and the second in the outer transmembrane helix close to the extracellular region of the channel. Moving to the brain circulation, we previously reported the close association (< 40 nm) of Kir2 channel subunits and Cav1 within caveolae structures in the membrane of brain vascular smooth muscle cells, a tandem arrangement that may enhance Kir2 channel mechanosensitivity (22).

Intriguingly, we recently demonstrated that simulated pressure using a hypoosmotic challenge, suppressed cerebral arterial smooth muscle Kir2 currents in whole-cell recordings, an effect that was entirely recovered by pre-incubation with actin-disrupting agents (i.e., latrunculin A and/or cytochalasin D). Disruption of caveolae-forming proteins similarly prevented suppression of smooth muscle Kir2 currents during cell-swelling. Moreover, immunofluorescence approaches revealed the expression of both scaffolding proteins, syntrophin and Cav1, in vascular smooth muscle and a proximity ligation assay highlighted the intimate structural association with Kir2.2 subunits (within 40 nm of one another), suggesting their participation in channel mechanosensing. These findings provided compelling evidence that Kir2 channel mechanosensitivity involves unique interactions with the cytoskeleton which are likely driven by scaffolding proteins.

### Cellular Pools of Kir Channels, Biomechanical Stimulus, and PIP_2_- vs. Cholesterol-Kir2 Interactions

Numerous studies have proposed vascular Kir2 channels as mechanosensitive components, primarily involved in the initial response of both pressure-induced vasoconstriction and flow-induced vasodilation of resistance arteries (21, 23, 143). Wu et at. (24) originally demonstrated that smooth muscle Kir2 channels serve as molecular mechanosensors, playing a critical role in the development of pressure-induced constriction (*myogenic tone*) of brain resistance arteries. Specifically, Kir2 currents recorded from native SMCs were potently suppressed during hypoosmotic challenge (swelling), a stimulus known to activate mechanically sensitive inward currents, leading to myogenic-like responses in the resistance vasculature (103, 154, 155). We recently proved and further expanded these findings by applying negative pressure through the patch pipette (conventional whole-cell configuration) via pneumatic transducer (-15 to -45 mmHg). This complementary approach to reduce cell stretching caused an evident increase in the smooth muscle Kir2 conductance (22), reinforcing the view of Kir2 channels as exquisite mechosensitive elements of the vascular cell membrane. These impressive observations may imply a dynamic interplay between Kir2 and pressure-sensitive transient receptor potential (TRP) channels to facilitate myogenic tone development (105, 156). Accordingly, greater Ba^2+^-induced constrictions (employed as an index of Kir activity) were observed when the arteries were exposed to low intravascular pressures, where the channel is most active. We further demonstrated that the lipid microenvironment surrounding Kir2 can impact the ability of the channel to respond to the pressure stimuli. In this framework, membrane cholesterol depletion prevented pressure-induced Kir2 inhibition *in vitro* and significantly augmented Ba^2+^-induced vasoconstriction in response to high intraluminal pressures. Together, these provocative data suggest that cholesterol-smooth muscle Kir interactions are crucial for hemodynamic sensing of pressure in the cerebral vasculature (22).

The strategic location of the vascular endothelium—at the interface between tissue and blood—functionally implies the involvement of the endothelial Kir2 channel population in flow-sensing. Supporting this hypothesis, a great body of studies employing a combination of *ex vivo* functional and *in vitro* electrophysiology approaches identified the endothelial shear-sensitive Kir2.1 channel as a major contributor of flow-induced dilation of resistance arteries (63, 157). Briefly, elevations in blood flow-associated shear stress initiates endothelial cell membrane hyperpolarization (∼ 2–6 mV in response to shear stresses of 1–5 dyn/cm^2^) by increasing the open probability of K^+^ channels—a response entirely blocked by micromolar concentrations of extracellular Ba^2+^ (a specific blocker of Kir2 channels) or Cs^+^ and/or completely abolished in mesenteric ECs freshly isolated from genetically modified mice lacking the Kir2.1 gene (heterozygous deletion of Kir2.1) (63, 113, 158). Moving to the brain vasculature, we demonstrated that fluid shear stress enhanced the density of native endothelial Kir currents, a response that was functionally translated into flow-induced dilation of cerebral resistance arteries as this response was prevented by intraluminal perfusion BaCl_2_ perfusion. We further showed a decrease of shear stress potentiation of endothelial Kir2 channel activity and a consequent abolition of flow-induced vasodilation of endothelium-intact cerebral resistance arteries following neomycin-induced membrane PIP_2_ depletion (22). These impressive findings identify PIP_2_ as a crucial driver in conferring shear stress sensitivity to the endothelial Kir2 channel population (22). Paradoxically, recent work from Levitan’s group revealed a marked reduction in the flow-induced activation of endothelial Kir2.1 channel activity during hypercholesterolemic conditions (159). These findings were reinforced using isolated arteries from an established mouse model of dyslipidemia-induced endothelial dysfunction (Apolipoprotein E-deficient mouse; apoE^–/–^) (160), in which depletion of membrane cholesterol content rescued flow-induced vasodilation in a Kir2.1-dependent manner (159). In line with these findings, the authors propose that endothelium dysfunction exhibited during hypercholesterolemia (and potential arteriosclerotic vascular disease) may be attributable to cholesterol-induced inhibition of Kir2.1 channels. Brain vascular endothelial Kir2 channels display less sensitivity to cholesterol (22), however, enhanced levels of this lipid in plasma during dyslipidemia may induce a dynamic reorganization of lipid content at the membrane level with an increase in cholesterol and possibly a drop in PIP_2_. This resulting lipid membrane profile would push adjacent endothelial Kir2 channels to reside in a preferred silent state, therefore rendering them insensitive to shear stress stimulation. Consistent with this notion, a previous study from Levitan’s group (159) and preliminary findings from our group (161) revealed a marked reduction in endothelial Kir2 channel activity and a consequent impairment of laminar flow-induced vasodilation in cerebral arteries isolated from two distinct models of dyslipidemia in mice. These observations have also been observed in humans, manifesting as impaired flow-induced vasodilation in hypercholesterolemic patients, thus suggesting cholesterol-induced suppression of Kir2.1 as a mechanism underlying endothelial dysfunction in this pathology (162).

## Concluding Remarks and Future Perspectives

**FIGURE 4:**
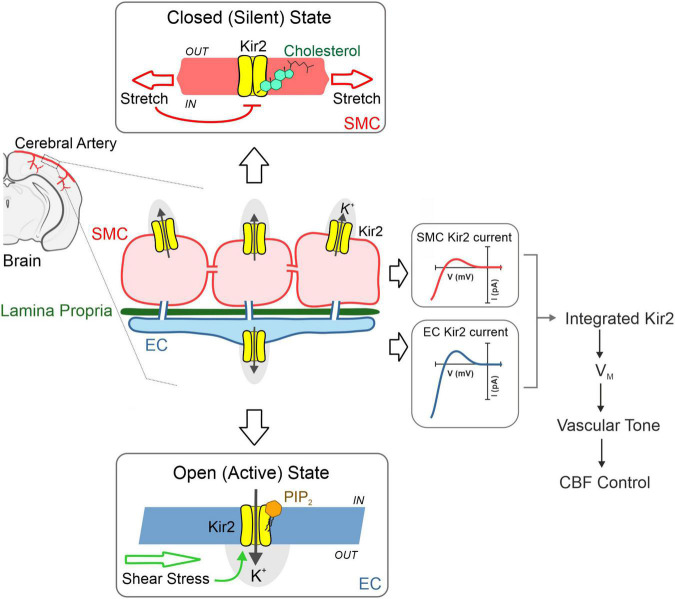
Cerebrovascular Kir2 channels and hemodynamic control. Schematic depiction explaining the modulation of two cellular populations of Kir2 channels, functionally expressed in smooth muscle and endothelium. Briefly, structural membrane lipids including PIP_2,_ and cholesterol represent primary regulators of Kir2 channels, with the former robustly stimulating endothelial Kir2, whereas the latter preferentially suppresses smooth muscle Kir2 activity. These lipid-Kir interactions help confer pressure and shear stress sensing, hemodynamic forces that diminish and enhance smooth muscle and endothelial Kir2 channel activity, respectively. These cellular channel populations dynamically interact to drive an integrated Kir2 current through which hemodynamic stimuli contribute to set V_*M*_, arterial tone and cerebral blood flow (CFB) control. Modified from Sancho et al. (22).

**FIGURE 5:**
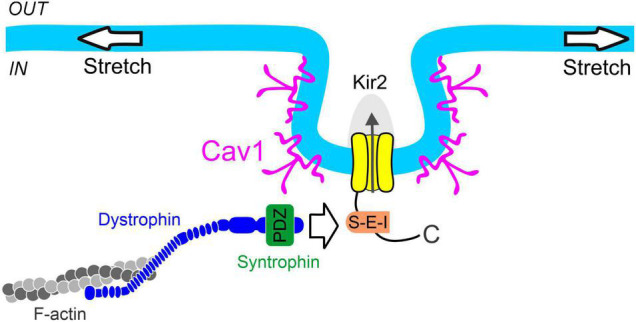
Proposed mechanism of smooth muscle Kir2 channel mechanosensitivity. Syntrophin binds to the C-terminal PDZ binding motif (S-E-I) of Kir2 channels residing in caveolae-like structures formed through the expression of caveolin-1 (Cav1). Dystrophin interacts with both syntrophin and filamentous actin (F-actin), providing a physical link between the Kir2 channel and cytoskeleton for force transmission. These structural components constitute a multiprotein signaling complex which confers pressure sensitivity to smooth muscle Kir2 channels.

Cerebral resistance arteries contain various cellular types including smooth muscle and endothelial cells—two intimately associated components that work in concert to set basal tone and control brain tissue perfusion. The plasma membranes of these vascular cells are constantly exposed to a range of biomechanical forces including membrane stretch (pressure) and shear stress (flow). These vascular cell types express functional Kir2 channels, and each cellular population is distinctly modulated by key lipid signaling molecules which can sense diverse hemodynamic forces. Particularly, Kir2 channels are uniquely modulated by membrane PIP_2_ and cholesterol depending on their cellular location (endothelium *vs*. smooth muscle); being these specific lipid-Kir2 channel interactions crucial to confer shear stress- and pressure sensitivity to the endothelial and smooth muscle Kir channel pools, respectively. These two cellular Kir2 populations have been proposed to actively interact to drive an integrated vascular Kir2 conductance designed to ultimately govern V_*M*_ and basal tone in the brain circulation (Figure 4) (22). The role of cytoskeleton and focal adhesion proteins is likely to be a major player in these hemodynamic responses. In this context, we recently proposed a model in which smooth muscle Kir2 mechanosensitivity is in part, conferred by a multiprotein complex comprising Kir2.2, syntrophin, actin, and Cav1 (Figure 5). It is also intriguing to consider the involvement of the cytoskeleton and diverse scaffolding proteins in the context of shear stress responsive endothelial mechanisms. In the short-term future, genetic manipulations at the level of stretch- and shear-sensitive ion channels will represent valuable tools to further study vascular mechanosensation, a fascinating area of research.

These mechanistic insights may provide important translational implications in the framework of dyslipidemia, a lipid disorder characterized by elevated plasma LDL-cholesterol levels. Given the endothelium anatomical location, ECs would be constantly exposed to higher levels of LDL-cholesterol, which in turn may impact lipid-Kir interactions and cause Kir2 channel stabilization to a preferred closed state. Therefore, under dyslipidemia a regulatory switch (PIP_2_ to cholesterol) would suppress endothelial Kir2 activity, impair shear stress-induced activation, and ultimately impact brain blood flow. This future area of research is intended to analyze the possible role of endothelial dysfunction as an early predictor of atherosclerosis. Undoubtedly, the translation of these observations to humans will be the basis for the development of accurate therapeutic avenues to treat patients at risk of early cardiovascular disease (163).

## Author Contributions

MS and JF wrote, designed the figures, and edited the manuscript. DGW edited the manuscript. All authors approved the submitted version.

## Conflict of Interest

The authors declare that the research was conducted in the absence of any commercial or financial relationships that could be construed as a potential conflict of interest.

## Publisher’s Note

All claims expressed in this article are solely those of the authors and do not necessarily represent those of their affiliated organizations, or those of the publisher, the editors and the reviewers. Any product that may be evaluated in this article, or claim that may be made by its manufacturer, is not guaranteed or endorsed by the publisher.
